# Transcriptomic and Metabolomic Profiles Provide Insights into the Red-Stipe Symptom of Morel Fruiting Bodies

**DOI:** 10.3390/jof9030373

**Published:** 2023-03-18

**Authors:** Chi Yang, Xiaoling Jiang, Lu Ma, Donglai Xiao, Xiaoyu Liu, Zhenghe Ying, Yaru Li, Yanquan Lin

**Affiliations:** Institute of Edible Mushroom, National and Local Joint Engineering Research Center for Breeding & Cultivation of Featured Edible Mushroom, Fujian Academy of Agricultural Sciences, Fuzhou 350014, China; yangchi@faas.cn (C.Y.);

**Keywords:** *Morchella*, red-stipe symptom, transcriptome, metabolome, tyrosine pathway

## Abstract

The cultivation of true morels (*Morchella* spp., Morchellaceae, Ascomycota) has rapidly expanded in recent years, especially in China. Red stipe is a symptom wherein the stipe of morel fruiting bodies becomes red–gray, resulting in the gradual death of the affected fruiting bodies. The impact of red-stipe symptom occurrence on the development and nutritional quality of morel fruiting bodies remains unclear. Herein, morel ascocarps with the red-stipe symptom (R) and normal (N), artificially cultivated in the Fujian Province of China, were selected for the transcriptome and metabolome analysis to study the physiological and biochemical responses of morel fruiting bodies to the red-stipe symptom. Transcriptome data revealed several differentially expressed genes between the R and N groups significantly enriched in the tyrosine, riboflavin, and glycerophospholipid metabolism pathways. Similarly, the differentially accumulated metabolites were mainly assigned to metabolic pathways, including tyrosine, the biosynthesis of plant secondary metabolites, and the biosynthesis of amino acids. Moreover, the transcriptome and metabolome data combination revealed that tyrosine metabolism was the most enriched pathway, which was followed by ATP-binding cassette (ABC) transport, alanine, aspartate, and glutamate metabolism. Overall, the integration of transcriptomic and metabolomic data of *M. sextelata* affected by red-stipe symptoms identified several important genes, metabolites, and pathways. These findings further improve our understanding of the mechanisms underlying the red-stipe symptom development of *M. sextelata* and provide new insights into how to optimize its cultivation methods.

## 1. Introduction

True morels (*Morchella* spp., Morchellaceae, Ascomycota) are well known for their unique flavor and health-promoting properties [1,2]. *Morchella* consumption has rapidly expanded in recent years owing to various biological functions and rich nutritional value [1,3,4,5]. The commercial demand for morels is increasing worldwide, despite their high price [6]. The genus *Morchella* contains numerous species, with more than 263 records in Index Fungorum (http://www.indexfungorum.org, accessed on 13 March 2023). To date, a total of 78 *Morchella* species have been discovered worldwide based on phylogenetically distinct characteristics [7]. Over the years, numerous attempts have been made to artificially cultivate morels due to the limited yield of naturally grown wild morels [8]. Nine cultivable species of morels are found in China, including *M. sextelata*, *M. eximia*, *M. exuberans*, *M. importuna*, *M. owneri*, *M. rufobrunnea*, *M. tomentosa*, *Mel-13*, and *Mel-21* [9,10]. Morel cultivation has achieved significant breakthroughs in China since 2012, with an area of 16,466 ha during the 2021–2022 season [11]. Meanwhile, morel biology has been recently extensively studied with important findings on its reproductive modes [7,12,13,14,15,16], nuclear and mitochondrial genome sequence and function [9,16,17,18,19,20,21], microbial community dynamics [22,23,24,25,26], nutrient metabolism [27], and fruiting body development [28,29,30,31].

Despite the rapid expansion of artificial morel cultivation in China, its cultivation industry is still facing several setbacks, including low spawn quality, spawn aging, environmental factors, mechanisms of exogenous nutrition, diseases, and management measures [5,7,11]. The large-scale, outdoor morel cultivation in an open environment infrastructure renders their ascocarps susceptible to pests and bacterial and fungal diseases [5]. Thus, preventing and controlling the disease spread is crucial for optimizing morel cultivation. It is estimated that 25% of the morel cultivation area has been affected by fungal infections, resulting in severe yield losses [11]. Several pathogenic fungi that infect morels have been identified, including *Cladobotryum protrusum* [32], *Diploöspora longispora* [33], the *Fusarium incarnatum*–*equiseti* species complex [34], *Paecilomyces penicillatus* [26,35], and *Lecanicillium aphanocladii* [36], have been identified. Recently, ITS-amplicon sequencing and microscopic examination were performed on morel ascocarp lesions from 32 sites in 18 provinces across China, revealing that *D. longispora* was a major cause of morel fungus diseases [11].

Although research on morel cultivation practices is still in its infancy, several studies have examined morel immune response to mycoparasites. Laccase was found to be involved in *Trichoderma aggressivum* toxin metabolism and enhanced resistance to green mold disease in *Agaricus bisporus* [37]. In *A. bisporus*, 17 simple sequence repeat markers have been associated with resistance to *Mycogone perniciosa* [38]. The genomic regions associated with dry-bubble resistance in *A. bisporus* were determined by quantitative trait loci (QTL) analysis of four traits related to resistance [39]. The genetic mechanisms of the interactions between *M. sextelata* and *P. penicillatus,* responsible for white mold disease in *Morchella* were studied using a dual RNA-Seq approach in both the host and the pathogen [40]. In infected *M. sextelata*, genes encoding for a chitin recognition protein, tyrosinases, a caffeine-induced death protein, Laccase-2, cytochrome P450s, and putative apoptosis-inducing proteins were upregulated, whereas cyclin was downregulated. Moreover, in *P. penicillatus,* several CAZymes involved in the host cell wall degradation were upregulated. Furthermore, 1-octen-3-ol was found to reduce the severity of white mold disease by suppressing *P. penicillatus* in the soil and improving morel yields [41].

In the spring of 2022, a disease severely affected morel yields, manifesting with red-stipe symptom that resulted in the cessation of fruit-body growth. Typically, the entire stalk is yellowish at the beginning of the disease; then, the color deepens and spreads from the base to form a distinct gray–black patch. The morels are visibly seen to lose viability, stop growing, and eventually rot. The disease can cause serious yield reduction or even the complete loss of morel mushrooms in a short period of time. Few researchers opine that the symptoms are caused by a bacterial pathogen [42], while another study identified the pathogen as *Fusarium nematophilum* [43]. Other researchers suggest that it is caused by insufficient nutrition during the low or high-temperature period, but this has not been confirmed and needs more experimental evidence. The most important and obvious symptom is that the stipe of morel fruiting bodies becomes red-gray, and the affected fruiting bodies gradually die. To this end, to unravel the physiological and biochemical responses of morel fruiting bodies to the red-stipe symptom, the dynamic changes of the affected *M. sextelata* in transcriptome and metabolome were determined. By integrating the transcriptomic and metabolomic information from the red (R) and normal (N) fruiting body groups, we identified a few key genes, metabolites, and pathways associated with the red-stipe symptom. Overall, our results provide novel insights into the metabolic mechanisms and nutritional composition changes underlying the red-stipe symptom of *M. sextelata*.

## 2. Materials and Methods

### 2.1. Sample Collection

**Figure 1:**
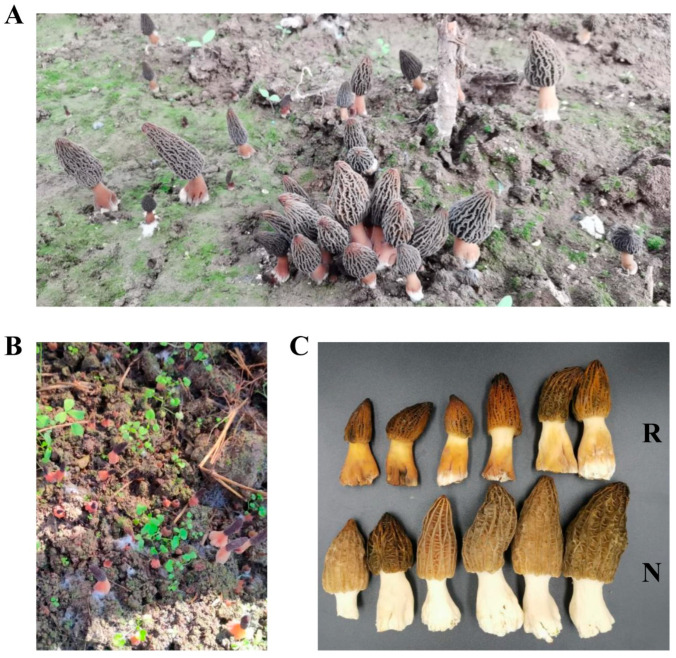
Samples of the red fruit body disease of *Morchella* sp. (**A**,**B**) Two examples of red fruit body disease Morels. (**C**) Samples used for analysis. Red group (R) referred to the samples with red stipe, while the normal group (N) referred to the samples with white stipe.

Fresh cultivated morels were collected from a farm (119.199865° E, 26.712312° N) in the Fujian province of China in March 2022 during the fruiting period. Two groups (R and N) of fresh morels were collected and quickly frozen in liquid nitrogen and then stored in a −80 °C deep freezer. The morphology of the cultivated fresh morels is shown in Figure 1A,B. The color of the fruiting body in the R group was changed to red or yellow, whereas that of the N group remained white (Figure 1C).

### 2.2. RNA Sequencing

Three samples from each N and R group were collected for transcriptome sequencing (three samples for each stage). Total RNA was extracted using the TRIzol reagent (Invitrogen, CA, USA), and their quality was assessed using the Bioanalyzer 2100 and RNA 1000 Nano LabChip Kit (Agilent, CA, USA) with RIN (RNA integrity number) >7.0. Poly-T magnetic beads were used twice to purify poly(A) RNA from total RNA (5 μg). Following purification, mRNA was fragmented with divalent cations at elevated temperatures and then reverse transcribed to create the final cDNA library with an average insert size of 300 bp (±50 bp). Thereafter, paired-end sequencing was performed on the Illumina Novaseq™ 6000 platform (LC Sciences, TX, USA) following the manufacturer’s protocol.

### 2.3. De Novo Assembly, Unigene Annotation, and Functional Classification

Adaptor-containing reads, low-quality bases, and undetermined bases were removed by Cutadapt [44]. Subsequently, sequence quality, including the Q20, Q30, and GC content of the clean data, was verified using FastQC (http://www.bioinformatics.babraham.ac.uk/projects/fastqc/ (accessed on 24 June 2022)). With Trinity 2.4.0 [45], a de novo transcriptome assembly was generated. All assembled unigenes were aligned against the sequences of the non-redundant (Nr) protein database (http://www.ncbi.nlm.nih.gov/ (accessed on 24 June 2022)), Gene Ontology (GO) (http://www.geneontology.org (accessed on 24 June 2022)), SwissProt (http://www.expasy.ch/sprot/ (accessed on 24 June 2022)), Kyoto Encyclopedia of Genes and Genomes (KEGG) (http://www.genome.jp/kegg/ (accessed on 24 June 2022)), and eggnog (http://eggnogdb.embl.de/ (accessed on 24 June 2022)) databases using DIAMOND [46] with an E-value threshold of <0.00001.

### 2.4. Differentially Expressed Unigene Analysis

To determine the unigene expression levels, Salmon [47] was used to calculate TPM (Transcripts Per Million) [48]. The differentially expressed unigenes were selected with a |log 2 (fold change)| > 1 at *p*-value < 0.05 cutoff using the R package edgeR [49]. GO enrichment analysis was carried out using the GOseq R package-based Wallenius non-central hyper-geometric distribution [50]. The KOBAS [51] software was used to test the statistical enrichment of DEGs in KEGG pathways [52,53,54].

### 2.5. Metabolite Extraction and Parameter Setting

The collected morel samples were thawed on ice, and metabolites were extracted using a 50% methanol buffer as described previously [55]. Pooled QC samples were prepared by combining 10 μL of each extraction mixture [56]. To identify differentially accumulated metabolites (DAMs) during the fruiting body development of the red-stipe symptom, we randomly analyzed six samples via liquid chromatography–mass spectrometry (LC–MS) as described previously [55]. An UltiMate 3000 HPLC (ThermoScientific, Waltham, MA, USA) was used for all chromatographic separations. The reversed-phase separation was conducted with an ACQUITY UPLC BEH C18 column (100 mm × 2.1 mm, 1.8 m; Waters, Wilmslow, UK). LC–MS was performed at a flow rate of 0.4 mL/min with solvent A (water containing 0.1% formic acid) and solvent B (acetonitrile containing 0.1% formic acid). Gradient elution was performed in the following order: 0–0.5 min, 5% solvent B; 0.5–7 min, 5–100% solvent B; 7–8 min, 100% solvent B; 8–8.1 min, 100%–5% solvent B; 8.1–10 min, 5% solvent B. The injection volume for each sample was 4 µL. The metabolites eluted from the column were detected with a high-resolution tandem mass spectrometer Q-Exactive (ThermoScientific). The AGC target of 3e6 was achieved by operating the Q-Exactive in both positive and negative ion modes. Precursor spectra were collected at a resolution of 70,000 from a range of 70–1050 m/z. The injection time was set at 100 ms, and the top-three configuration was selected to acquire data in the DDA mode. To hit an AGC target of 1e5 with a maximum injection time of 80 ms, fragment spectra were collected at a resolution of 17,500.

### 2.6. Metabolite Identification and Quantification

MSConvert was used to transform the LC–MS raw data into the mzXML format, which was then processed using XCMS, CAMERA, and the metaX toolbox, which was implemented in the R software. Each ion was identified by its retention time (RT) and m/z data. The online KEGG (http://www.kegg.jp/ (accessed on 24 June 2022)) and HMDB (http://www.hmdb.ca/ (accessed on 24 June 2022)) databases were used to annotate metabolites. The peak data intensities were further preprocessed using metaX (http://metax.genomics.cn/ (accessed on 24 June 2022)). The features detected in <50% of the QC samples or 80% of the biological samples were eliminated, and further improvement was made using imputed missing values with the k-nearest neighbor algorithm. Pre-processed data were subjected to principal component analysis (PCA) to detect outliers and evaluate batch effects. Student *t*-tests were conducted to detect differences in metabolite concentrations between the R group and N group. A false discovery rate (FDR; Benjamini–Hochberg) was used to adjust *p* values for multiple testing corrections. metaX was used to conduct supervised partial least squares-discriminant analysis (PLS-DA) to discriminate between groups. A VIP cut-off value of 1.0 was used to select important features.

### 2.7. Identification of Key Pathways and Validation of Gene Expression

To assess pathway enrichment, co-joint analyses were performed on genes DEGs and DAMs. To assess the relationship between the transcriptome and metabolome data, the common KEGG pathways were identified using Venn diagram analysis based on the functional annotation of DEGs and DAMs. qRT-PCR (Quantitative real-time polymerase chain reaction) was used to analyze gene expression, as previously described [57]. Total RNA was isolated using the TRIzol reagent (Invitrogen) and reverse transcribed using the PrimeScriptTM II First Strand cDNA Synthesis Kit (Takara, Japan). Each gene was assessed in duplicate, and the obtained Ct values were normalized using the housekeeping gene *ACT1* [58]. NCBI primer-BLAST was used for primer design (https://www.ncbi.nlm.nih.gov/tools/primer-blast (accessed on 15 October 2022)). The primers used in this study are described in Supplementary Table S1.

qRT-PCR was performed in 96-well optical plates using the CFX Connect Real-Time System (Applied with Bio-Rad, Hercules, CA, USA). The reaction mixture contained 2.0 μL cDNA, 0.5 μL of each primer (10 μM), 10.0 μL 2× SYBR Premix Ex Taq, and ddH_2_O to make a final volume of 20 μL. The thermal cycling conditions were as follows: the reaction mixture was heated at 95 °C for 1 min and then subjected to 40 cycles of 10 s at 95 °C, which was followed by 34 s at 60 °C and 60 s at 60 °C. Three biological and technical replicates were used.

## 3. Results

### 3.1. Transcriptome Sequencing and Functional Annotation

The transcriptome sequencing data analysis results are presented in Supplementary Table S2. A total of 241,412,342 high-quality clean reads were obtained from six samples, corresponding to 33.51 GB of clean data. The ITS sequence of sample identified by similarity searches in GenBank showed 100% identity to the *M. sextelata* strain S4 (GenBank accession number: MK616098.1). So, the *M. sextelata* genome (https://www.ncbi.nlm.nih.gov/genome/86229?genome_assembly_id=1702542 (accessed on accessed on 24 June 2022)) was selected for sequence alignment. However, the overall alignment rate was only 61.51%. Hence, a de novo transcriptome assembly was performed (data not shown). A total of 22,969 unigenes with an N50 length of 1585 bp were de novo assembled from high-quality clean reads (Supplementary Table S3). The annotation results of all the unigenes are shown in Supplementary Tables S4–S6. Most of the unigenes (74.68%) were hit to *Morchella*, while only 6.32% genes hit to *Lentinula edodes* (Supplementary Table S5 and Figure S1).

### 3.2. Global Changes in Gene Expression

**Figure 2:**
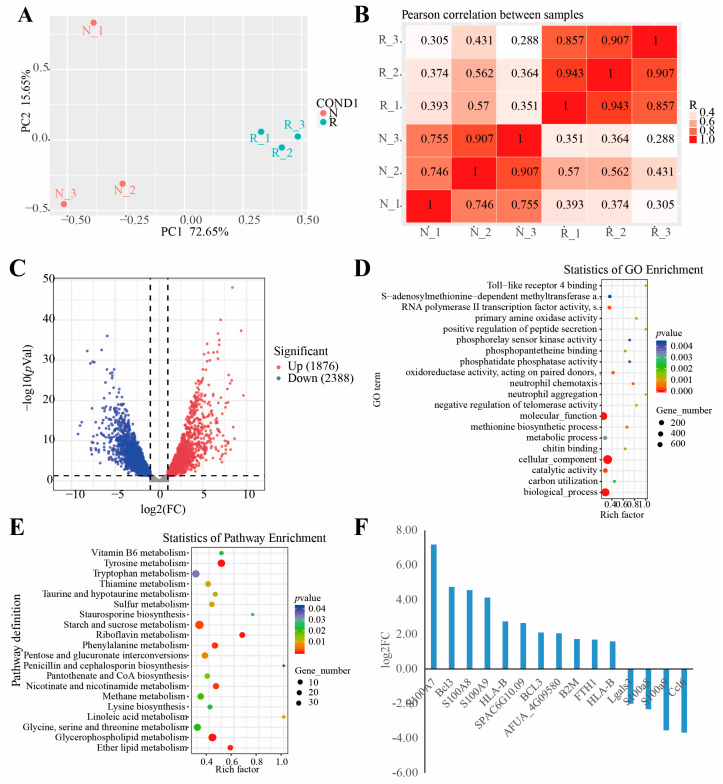
Results of RNA-seq. (**A**) Principal component analysis (PCA) of the variance-stabilized estimated raw counts of differentially expressed genes. (**B**) Correlation heat map. (**C**) Volcano plot of gene expression levels. (**D**) GO functional enrichment analysis. (**E**) KEGG functional enrichment analysis. (**F**) Fifteen DEGs from the immune-related GO terms; the *y*-axis shows the fold change expression in R group compared with N group.

Principal component analysis revealed that the samples were clustered based on the N and R groups (Figure 2A). The first (PC1) and second (PC2) principal components explained 72.65% and 15.65% of the total variation across the data set. A plot of PC1 and PC2 scores showed a clear separation between the groups across PC1. Meanwhile, the samples from each group were clustered together (Figure 2B). A total of 1876 and 2388 genes were significantly up- and downregulated, respectively, in the R group compared to the N group (Figure 2C and Supplementary Table S7). *TRINITY_DN14301_c2_g8* was the most upregulated (log2 FC = 9.64; Supplementary Table S7). However, the function of this gene is unknown. Interestingly, genes encoding chitin recognition protein (*TRINITY_DN5661_c0_g1*), an allergen Asp f 15 precursor (*TRINITY_DN13718_c0_g4*), and cyclin-dependent protein kinase (*TRINITY_DN14130_c2_g15*) were among the DEGs (Supplementary Table S7), and they have been previously reported to be involved in immune responses of mushrooms to pathogenic fungi [40].

The DEGs were further subjected to GO enrichment analysis, and 151 GO terms were identified (*p* < 0.05) (Figure 2D and Supplementary Tables S8 and S9). The most strikingly enriched GO terms in the biological process category were “neutrophil chemotaxis (GO: 0030593),” “methionine biosynthetic process (GO: 0009086),” and “negative regulation of telomerase activity (GO: 0051974)”. In the molecular function category, “catalytic activity (GO: 0003824),” “RNA polymerase II transcription factor activity (GO:0000981),” and “oxidoreductase activity, acting on paired donors, with incorporation or reduction of molecular oxygen (GO: 0016705)” were the most significantly enriched terms. Whereas terms associated with “TRC complex (GO: 0072380),” “hyphal cell wall (GO: 0030446),” and “90S preribosome (GO: 0030686)” were significantly enriched in the cellular component category. Overall, 15 DEGs, including 11 upregulated genes and 4 downregulated genes, were annotated to the immune-related GO terms (Figure 2F and Supplementary Table S8). Additionally, all the DEGs were mapped to the KEGG database, and 23 significantly enriched pathways were identified (*p* < 0.05) (Supplementary Table S10). Among them, “tyrosine metabolism (map00350),” “riboflavin metabolism (map00740),” and “glycerophospholipid metabolism pathways (map00564)” were the most significantly enriched (Figure 2E and Supplementary Table S11).

### 3.3. Metabolome Profiling

**Figure 3:**
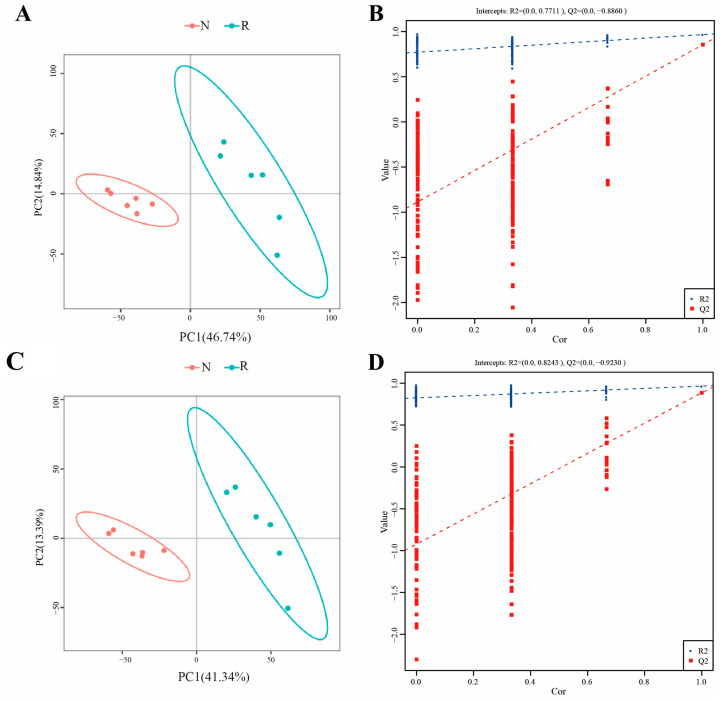
OPLS-DA analysis among samples. (**A**) OPLS-DA score of negative mode, (**B**) OPLS-DA validation of negative mode, (**C**) OPLS-DA score of positive mode, (**D**) OPLS-DA validation of positive mode.

To determine the global metabolite profiles in the R and N groups, LC–MS metabolome analysis was performed. As shown in Supplementary Table S12, 4, 3829 and 3856 metabolites were identified using the POS (positive) and NEG (negative) models, respectively, among which 1894 and 1417 were annotated. Orthogonal projections to latent structures-discriminant analysis revealed that the metabolic features formed distinct clusters (six samples in each group) when comparing the R and N groups in both negative (Figure 3A,B) and positive ion modes (Figure 3C,D).

**Figure 4:**
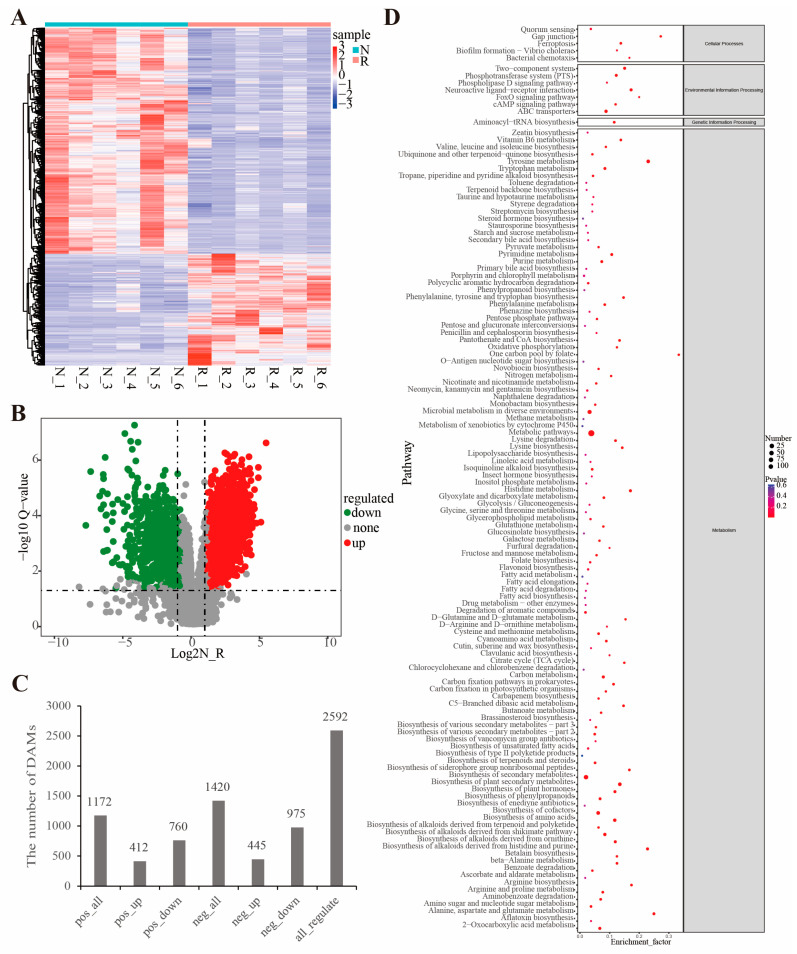
Differentially accumulated metabolites between R group and N group. (**A**) The heat map showing the differentially accumulated metabolites between R group and N group (the red and blue colors indicate the pathways enriched by the upregulated and downregulated metabolites, respectively). (**B**) Volcano plot of metabolite levels. (**C**) Statistical results of DEGs. (**D**) KEGG functional enrichment analysis.

Based on the identified metabolites quantitation, there were DAMs between the different groups. They included 412 upregulated and 760 downregulated in the POS mode and 445 upregulated and 975 downregulated in the NEG mode (Figure 4A–C, and Supplementary Table S11). Based on functional analysis (Figure 4D and Supplementary Table S13), 74 KEGG pathways (*p* < 0.05) were significantly enriched among these DAMs (Supplementary Table S14). The most enriched pathways in the metabolism category were “Tyrosine metabolism (map00350),” “biosynthesis of plant secondary metabolites (map01060),” and “biosynthesis of amino acids (map01230)”. “Ferroptosis (map04216),” “gap junction (map04540),” and “bacterial chemotaxis (map02030)” were among the enriched pathways in the cellular processes category; “neuroactive ligand-receptor interaction (map04080),” “ABC transporters (map02010),” and “two-component system (map02020)” were enriched in the environmental information processing category; and “aminoacyl-tRNA biosynthesis (map00970)” was enriched in the genetic information processing category. Among them, the “ferroptosis (map04216)” pathway was shown to affect the immune cell number and function and trigger a range of inflammatory or specific responses [59].

### 3.4. Identification of Key Pathways and Validation of Gene Expression

**Figure 5:**
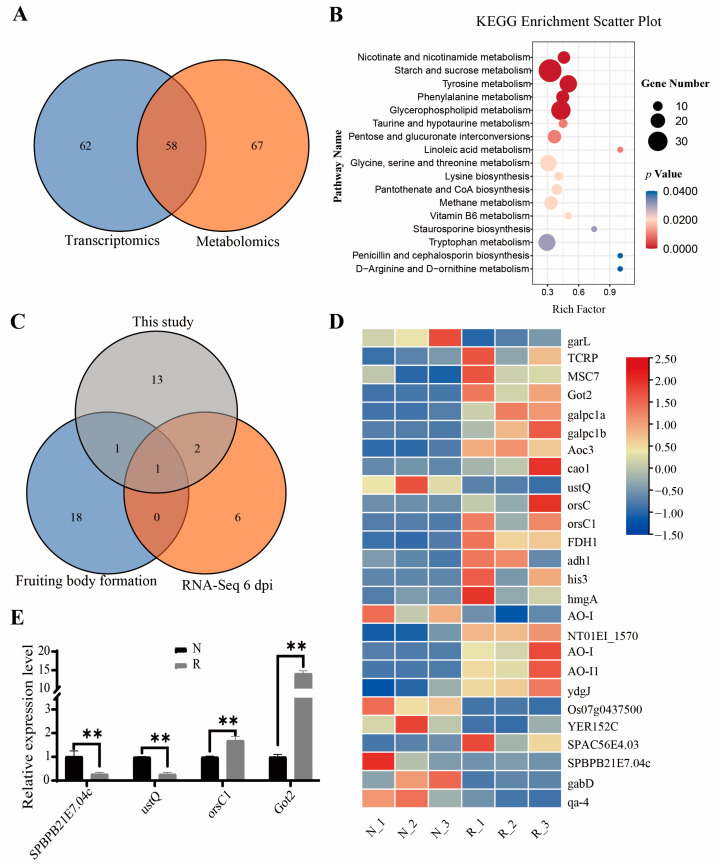
Comprehensive analysis of metabolome and transcriptome. (**A**) Venn diagram of pathways annotated in metabolome and transcriptome. (**B**) KEGG enrichment analysis. (**C**) Venn diagram of pathways among two previous studies [29,40] and this study. (**D**) Heat map of DEGs in tyrosine metabolism pathway, (E) Relative expression level of DEGs. The X axis contains the gene codes of DEGs and the Y axis contains the relative expression level of DEGs. Data were presented as mean ± SD. ** *p*-value < 0.01.

There were 58 pathways identified in both the metabolome and transcriptome profiling, among which 17 pathways were significantly enriched (*p* < 0.05) in the R group compared with the N group (Figure 5A,B and Supplementary Table S15). The three most enriched pathways were “nicotinate and nicotinamide metabolism (map00760),” “starch and sucrose metabolism (map00500),” and “tyrosine metabolism (map00350).” There were five amino acid metabolism-related pathways, two carbohydrate metabolism pathways, and five metabolism pathways significantly enriched in the R group. Overall, 257 out of 748 DEGs and 175 out of 189 DAMs were enriched in the combined transcriptome and metabolome analysis (Supplementary Tables S16 and S17). Compared to *Morchella* response to *P. penicillatus*, similarly determined using a dual RNA-Seq approach [40], “tryptophan metabolism (map00380)” and “pantothenate and CoA biosynthesis (map00770)” were also identified in our study. When compared to the *Morchella* fruiting bodies and mycelium metabolites based on widely targeted metabolomic analyses [29], only “glycerophospholipid metabolism (map00564)” was common in our study. Most interestingly, “tyrosine metabolism (map00350)” was the only pathway identified in both the above studies and our study (Figure 5C and Supplementary Table S18).

In total, 26 DEGs were identified belonging to the tyrosine metabolism pathway (Figure 5D and Supplementary Table S19), including three di-copper center-containing proteins, three PLP-dependent transferases, two class I glutamine amidotransferase-like proteins, two copper amine oxidases, and others. Four genes in the tyrosine metabolism pathway were selected for further validation using qPCR, including two downregulated genes, S-adenosyl-L-methionine-dependent methyltransferase (*TRINITY_DN7353_c0_g1*) and di-copper center-containing protein 1 (*TRINITY_DN12464_c0_g1*), and two upregulated genes, di-copper center-containing protein 3 (*TRINITY_DN14293_c1_g13*) and aminotransferase class I and II (*TRINITY_DN693_c0_g1*) (Supplementary Table S19). As shown in Figure 5E, all four genes exhibited similar expression patterns to the RNA-Seq results.

## 4. Discussion

Morel cultivation has expanded rapidly worldwide because of its low labor intensity, short production cycle, and its nutritional properties and health benefits [5]. However, morel cultivation in the field increases their susceptibility to diseases and pests, which is the most important cause of reduced yields and crop failure. More importantly, the climate in the primary morel-producing areas in China is warm and humid, which favors the spread of various insects and pests [60]. The occurrence and features of epidemics regarding *Morchella* cultivation have been previously studied. ITS amplicon sequencing and microscopic examination of morel ascocarp lesions were performed simultaneously at 32 sites in 18 provinces across China. It was found that *D. longispora* was the major culprit of morel fungal diseases [11]. *Lecanicillium aphanocladii* [36] and *Clonostachys rosea* [61] are the causal agents of rot disease *M. sextelata*, and *P. penicillatus* causes the white mold disease [35]. *M. sextelata* defense mechanisms against *P. penicillatus* were studied by dual host-pathogen RNA-Seq analysis, and 313 DEGs were identified [40]. The results revealed that programmed cell death in *M. sextelata* was triggered by *P. penicillatus* infection via the upregulation of a chitin recognition protein, a caffeine-induced death protein, a putative apoptosis-inducing protein, and cyclin downregulation. Moreover, 1-octen-3-ol, a volatile mushroom compound with broad-spectrum antimicrobial activities, was shown to control the disease caused by *P. penicillatus* [41]. However, limited studies have focused on the mechanisms underlying red-stipe symptom occurrence.

After the red-stipe symptom appearance, the ascocarp becomes sticky and malodorous with a red and withered stipe. Mushroom farmers are desperate when morels are affected by the red-stipe symptom disease. Studies have revealed that the red-stipe symptom could be caused by *Fusarium nematophilum* [43] and bacterial infection [42]. No matter the causes of red-stipe symptoms, the stipe of morel fruiting bodies becomes red-gray, and the affected fruiting bodies gradually die. To uncover gene expression changes as a result of red-stipe symptoms in morels, RNA-Seq was implemented. A total of 1876 and 2388 genes were significantly up- and downregulated in the R group compared to the N group, respectively (Figure 2C and Supplementary Table S3), which was much higher than that the DEGs reported in a previous study on the interaction between *M. sextelata* and *P. penicillatus* (313 DEGs). This discrepancy may be caused by the difference in data processing, as we used de novo transcriptome assembly while the authors of the previous study mapped the reads to the *M. sextelata* reference genome. Usually, a de novo assembly method can identify a higher number of DEGs. For example, 12,561 unigenes were identified as DEGs in the early fruiting body-formation stage compared to the mycelium stage, including 9215 upregulated and 3346 downregulated unigenes [30]. Before we used a de novo assembly in our study, we identified the morel sample species based on their ITS sequence. The ITS sequences queried using a similarity search in GenBank exhibited 100% identity to the *M. sextelata* strain S4 (GenBank accession number: MK616098.1). Thus, the genome of *M. sextelata* (https://www.ncbi.nlm.nih.gov/genome/86229?genome_assembly_id=1702542 (accessed on 24 June 2022)) was selected for alignment based on ITS identity. However, the overall alignment rate was only 61.51% (data not shown). Because most of the unigenes hit against the NR database belonged to *Morchella conica* (74.68%), we also mapped the reads to the genome of *Morchella conica* (https://ftp.ncbi.nlm.nih.gov/genomes/genbank/fungi/Morchella_conica/latest_assembly_versions/GCA_003790465.1_Morco1/ (accessed on 12 March 2023)), and the overall alignment rate was only about 20% (data not shown). Hence, we performed de novo transcriptome assembly. We also mapped the raw data to the *F. nematophilum* genome, resulting in an overall alignment percentage of less than 0.1%. Therefore, we can conclude that the symptoms of the samples we collected were not caused by *F. nematophilum.*

The most obvious red-stipe symptom phenotype is the color change. Plants have three main types of pigments: anthocyanins, carotenoids, and flavonoids [62]. Among the DEGs (Supplementary Table S3), one gene encoding for a leucoanthocyanidin dioxygenase (*TRINITY_DN2756_c0_g1*) was upregulated (log2 FC = 3.21; Supplementary Table S3) and has been associated with the red color phenotype in plants [63,64,65]. Leucoanthocyanidin dioxygenase is one of the flavonoid pathway dioxygenases catalyzing the formation of anthocyanidins from leucoanthocyanidins. Furthermore, β, β-carotene 15,15′-dioxygenase (*TRINITY_DN14050_c0_g12*) and β-apo-4′-carotenal oxygenase (*TRINITY_DN14186_c2_g21*) were upregulated in the R group (log2 FC = 1.95 and 1.43, respectively). A flavonoid biosynthesis pathway (GO: 0009813) gene, UDP-glycosyltransferase/glycogen phosphorylase (*TRINITY_DN13177_c0_g1*), was downregulated (log2 FC = −2.24) in the R group. Additionally, two upregulated genes (*TRINITY_DN14050_c0_g12* and *TRINITY_DN14186_c2_g21*) are involved in carotenoid biosynthesis (log2 FC = 1.95 and 1.43; Supplementary Table S3). These genes may therefore contribute to the red ascocarp color.

Based on untargeted metabolomic analyses (Supplementary Table S8), four flavonoids (neg-4.348_302.07894, neg-3.598_306.07385, neg-3.445_306.074, and pos-0.972_306.07537) and one 2-arylbenzofuran flavonoid (neg-5.146_580.31254) had a significantly higher concentration in the R group compared to the N group. In contrast, two flavonoids (pos-3.209_419.09922 and neg-3.706_366.00386), one 2-arylbenzofuran flavonoid (pos-3.881_376.16569), and three isoflavonoids (pos-1.351_352.09329, neg-0.948_352.09388, and neg-4.28_408.05163) had a lower concentration in the R group compared to the N group. In addition to flavonoids, four metabolites in the “cinnamic acids and derivatives” category were significantly higher in the R group compared to the N group. These results may pinpoint the differentially regulated genes and metabolites underlying the red stipe phenomenon.

The immune response system is important for host protection against pathogenic fungi. During the biotrophic infection stage, *Magnaporthe oryzae* suppresses host immune responses by triggering an oxidative burst, resulting in plant cell death [66]. A gene (*TRINITY_DN14093_c0_g1*) encoding for programmed cell death protein 6 was upregulated in the R group (log2 FC = 1.53; Supplementary Table S3). This was consistent with previous findings suggesting that *P. penicillatus* was suppressed by *M. sextelata* via programmed cell death [40]. At the same time, the GO term “oxidoreductase activity, acting on paired donors, with incorporation or reduction of molecular oxygen (GO:0016705)” was significantly enriched by the DEGs (Supplementary Table S4). Several genes involved in immune responses were differentially expressed in *P. penicillatu* infected *M. sextelata* [40]. According to our RNA-Seq results, 15 DEGs annotated to immune-related GO terms, including 11 upregulated and four downregulated genes in the R group (Figure 2F and Supplementary Table S3). Furthermore, genes encoding a chitin recognition protein (*TRINITY_DN5661_c0_g1*), an allergen Asp f 15 precursor (*TRINITY_DN13718_c0_g4*), and a cyclin-dependent protein kinase (*TRINITY_DN14130_c2_g15*) were identified among the DEGs (Supplementary Table S3), and they have been reported to be involved in *M. sextelata* immune responses against *P. penicillatus* [40].

Autophagy is an essential and conserved self-eating process that cells perform to allow the degradation of intracellular components [67]. Autophagy has been observed during the development, secondary metabolism, and infection of fungi [68,69,70]. Based on our RNA-seq results (Supplementary Table S3), 27 DEGs were enriched in autophagy-related KEGG pathways (map04136 and map04138). Importantly, three genes (TRINITY_DN14616_c1_g8, TRINITY_DN14211_c0_g4, and TRINITY_DN12413_c0_g3) encoding autophagy-related proteins were significantly differentially expressed. Although no autophagy-related KEGG pathway was enriched in the red-stipe symptom morel DAMs, certain metabolites were associated with autophagy, including mannitol (neg-1.083_182.07879 and neg-0.884_182.07874) and glutamine (pos-0.85_146.06874, pos-3.284_146.06897, pos-1.024_146.06877, neg-1.007_146.06889, and neg-0.821_146.06887). Thus, these metabolites are potentially associated with the red-stipe symptom of morels, which is consistent with a previous report that identified that autophagy was important in post ripeness and brown film formation in *Lentinula edodes* [71]. To maintain cell integrity, the cell wall serves as the primary barrier against the external environment [72]. Overall, 12 DEGs were identified belonging to the fungal-type cell wall (GO:0009277) GO term, including five upregulated and seven downregulated genes in the R group (Supplementary Table S3). In fungi, melanin, deposited in the cell wall and cytoplasm, is produced via tyrosinases, laccases, catecholases, and the polyketide synthase pathway [73]. According to our RNA-Seq results, the melanin biosynthetic process (GO:0042438) GO term was enriched (*p* < 0.05), including three upregulated genes (*TRINITY_DN15099_c0_g1*, *TRINITY_DN14263_c0_g25*, and *TRINITY_DN14239_c1_g11*) (Supplementary Table S5). Meanwhile, the laccase-2 gene (*TRINITY_DN16832_c0_g1*) was upregulated (log2 FC = 6.84) in the R group compared to the N group (Supplementary Table S3). Laccase-2 enhanced the resistance of *A. bisporus* against *T. aggressivum* toxin [37] and was upregulated in *M. sextelata* infected by *P. penicillatus* [40]. However, further functional or time series studies are required to verify the functions of these DEGs.

The most enriched metabolic pathway In the *Morchella* fruiting bodies and mycelium DAMs was that of tyrosine [29]. Tyrosine metabolism is mainly involved in different phenolic metabolite biosynthesis, and it indirectly affects the umami flavor of *Morchella*, exhibiting a vital role in the fruiting body development. ABC transporters and tyrosine metabolism were enriched in the DEGs between the *M. conica* mycelia and the fruiting body [24]. Tyrosine metabolism was also enriched in the *M. sextelata* response to *P. penicillatus* [40] and the red-stipe symptom (Figure 5C and Supplementary Table S13). Genes encoding browning-related enzymes in the *F. filiformis* genome are involved in tyrosine metabolism and phenylpropanoidbiosynthesis [74]. These results indicated that the tyrosine metabolism pathway is important for *Morchella* growth and development and resistance to pathogens. In the RNA-Seq results, 26 DEGs in the tyrosine metabolism pathway were queried, and the expression levels of four of these genes were validated using qRT-PCR (Figure 5D,E and Supplementary Table S14). Based on the metabolome profiling, 28 metabolites belonging to the tyrosine metabolism pathway were significantly accumulated, of which fumaric acid, vanillylmandelic acid, l-dopachrome, homovanillic acid, liothyronine, 4-hydroxyphenylacetic acid, and 2,5-dihydroxybenzaldehyde were upregulated in the R group compared to the N group (Supplementary Table S8). All of the above results indicate that the tyrosine metabolism pathway is key for red-stipe symptom development.

## 5. Conclusions

This is the first study implementing comparative transcriptomic and metabolomic analyses of *M. sextelata* fruiting bodies exhibiting the red-stipe symptom. Numerous important genes, metabolites, and pathways were identified associated with the red-stipe symptom development of *M. sextelata*. These findings have revealed new aspects of the mechanisms underlying the red-stipe symptom development and provide new insights into optimizing the morel cultivation practices.

## Data Availability

The data of RNA-seq in this study have been deposited in NCBI’s Gene Expression Omnibus (GEO) under accession number GSE212851. Metabolomic data have been deposited in the EMBL-EBI MetaboLights database (DOI:10.1093/nar/gkz1019, PMID:31691833) with the identifier MTBLS5913. The complete dataset can be accessed here https://www.ebi.ac.uk/metabolights/MTBLS5913 (accessed on 9 September 2022).

## Supplementary Materials

The following supporting information can be downloaded at: https://www.mdpi.com/article/10.3390/jof9030373/s1, Figure S1: Species distribution of NR annotation results of the *Morchella* transcripts; Table S1: Primers for the selected DEGs; Table S2: Sequencing data Statistics; Table S3: Trinity assembly results; Table S4: Annotation results of the Unigenes; Table S5: Gene annotation result using NCBI non-redundant protein database; Table S6: Functional annotation of unigenes in the NR, Swiss-prot, Pfam, COG, GO, and KEGG databases; Table S7: All DEGs in red-stipe symptom; Table S8: GO annotation of DEGs; Table S9: GO enrichment of DEGs; Table S10: KEGG annotation of DEGs; Table S11: KEGG enrichment of DEGs; Table S12: Statistics of identified metabolites; Table S13: All DAMs in red-stipe symptom; Table S14: KEGG enrichment DAMs; Table S15: KEGG pathways enriched both in transcriptomic and metabolomic profiles; Table S16: DEGs enriched in the combined analysis of transcriptome and metabolome; Table S17: DAMs enriched in the combined analysis of transcriptome and metabolome; Table S18: Pathways among two previous studies [29,40] and this study; Table S19: Summary of the DEGs in tyrosine metabolism pathway.
