# Supplementary material for: Transcriptomic and Metabolomic Profiles Provide Insights into the Red-Stipe Symptom of Morel Fruiting Bodies
Source: J Fungi (Basel). 2023 Mar 18;9(3):373. doi: 10.3390/jof9030373 (PMC10058789; doi:10.3390/jof9030373)
Supplement: Supplementary file 1 [file jof-09-00373-s001.zip › Supplementary Figures.pdf]

Supplementary Information

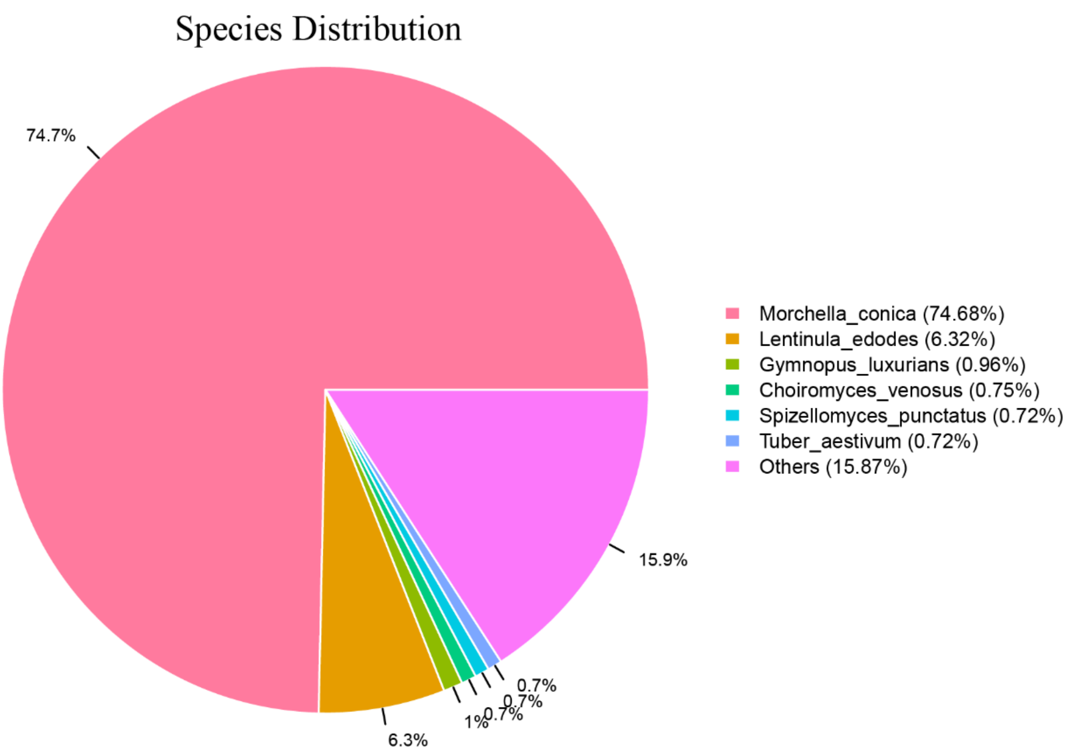

Supplementary Figure S1. Species distribution of NR annotation results of the *Morchella* transcripts.

Table S1. Primers for the selected DEGs.

Table S2. Sequencing data Statistics.

Table S3. Trinity assembly results.

Table S4. Annotation results of the Unigenes.

Table S5. Gene annotation result using NCBI non-redundant protein database.

Table S6. Functional annotation of unigenes in the NR, Swiss-prot, Pfam, COG, GO, and KEGG databases.

Table S7. All DEGs in red-stipe symptom.

Table S8 GO annotation of DEGs.

Table S9. GO enrichment of DEGs.

Table S10. KEGG annotation of DEGs.

Table S11. KEGG enrichment of DEGs.

Table S12. Statistics of identified metabolites.

Table S13. All DAMs in red-stipe symptom.

Table S14. KEGG enrichment DAMs.

Table S15. KEGG pathways enriched both in transcriptomic and metabolomic profiles.

Table S16. DEGs enriched in the combined analysis of transcriptome and metabolome.

Table S17. DAMs enriched in the combined analysis of transcriptome and metabolome.

Table S18. Pathways among two previous studies [29,40] and this study.

Table S19. Summary of the DEGs in tyrosine metabolism pathway.
