# Supplementary figures and images for: Transcriptomic and Metabolomic Profiles Provide Insights into the Red-Stipe Symptom of Morel Fruiting Bodies
Source: J Fungi (Basel). 2023 Mar 18;9(3):373. doi: 10.3390/jof9030373 (PMC10058789; doi:10.3390/jof9030373)

# Species Distribution

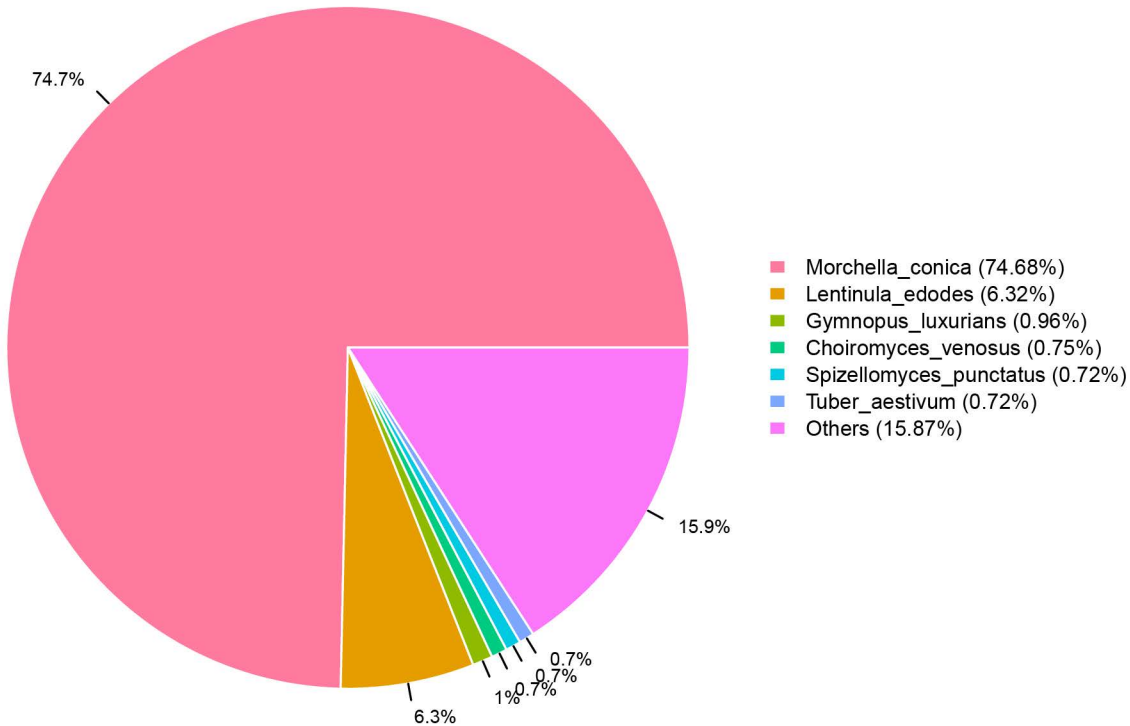

Supplement: Supplementary file 1 [file jof-09-00373-s001.zip › Fig S1gene_blast_species.pdf]
